# Could Management of Blood Pressure Prevent Dementia in the elderly?

**DOI:** 10.1186/s40885-019-0135-7

**Published:** 2019-12-10

**Authors:** Masaki Mogi

**Affiliations:** Department of Pharmacology, Ehime University, Graduate School of Medicine, Matsuyama, Japan

**Keywords:** Hypertension, Mixed dementia, Renin-angiotensin system, Blood brain barrier, Vascular degeneration

## Abstract

**Background:**

Hypertension is one of the most relevant risk factors in vascular aging, stroke and vascular dementia (VD). In the elderly, the prevalence of mixed dementia, which consists of Alzheimer’s disease (AD) and VD, is increased. Moreover, disorders of blood vessels are reported to be involved in the onset and progression of AD. Thus, hypertension generally plays an important role in dementia overall.

**Main Text:**

Mid-life hypertension is reported to be related to the incidence of dementia, but it is reported that antihypertensive treatment in aged people cannot prevent the onset and progression of dementia. The renin-angiotensin system (RAS) is deeply involved in not only hypertension but also lifestyle-related diseases, and may contribute to the pathological mechanism in dementia; thus, RAS regulation is expected to prevent dementia. Small vessel structural changes in lifestyle-related diseases may play a role in dementia in the elderly.

**Conclusion:**

Here, we discuss the role of blood pressure elevation in dementia and the therapeutic possibility of antihypertensive treatment against dementia.

## Background

Life expectancy is increasing worldwide. Especially, Asian countries such as Japan and South Korea face the serious problem of a longer lifespan. In South Korea, prolongation of life will be the largest worldwide, with life expectancy set to hit 90 years by 2030 [1]. We are now venturing into an unknown world in which life expectancy is over 90 years.

A more serious problem is the increase in people with dementia. The World Health Organization reports that dementia cases have tripled worldwide [2]. Especially in Asia, people with dementia will reach 70 million by 2050. In South Korea, the number of demented people is expected to double in ten years. On the other hand, the number of demented people will reach 7 million by 2025 in Japan. Unarguably, the demented population will become a severe worldwide problem in the near future.

However, new Alzheimer’s disease drugs have not emerged over the last decade. A review [3] reported that in 2010, four drugs, acetylcholine esterase inhibitors such as donepezil, rivastigmine, galantamine, and a N-methyl-d-aspartate (NMDA) receptor antagonist, memantine, were currently used drugs, but others had been developed. Although many clinical and experimental studies have been performed, however, the situation is unchanged in 2019. Moreover, treatment for Alzheimer’s disease using these four kinds of drugs is not radical. So, what should we do to overcome this serious problem? Potential brain mechanisms to be utilized in preventive strategies against dementia are reported in the Lancet [4]. According to the report, multi-domain lifestyle intervention such as increasing brain cognitive reserve, reducing brain damage, and reducing brain inflammation is necessary to prevent dementia. Vascular protection is involved in the reduction of brain damage. The American Heart Association also recommends increasing seven cardiovascular health metrics that people can improve through lifestyle changes, including maintaining untreated blood pressure below 120/80 mmHg [5]. A three-city study indicated that higher levels of such metrics were associated with lower incidence of dementia in 6626 elderly more than 65 years old [6], indicating that vascular protection via appropriate blood pressure management is a recommended lifestyle modification to prevent dementia.

Epidemiological studies indicate potentially modifiable risk factors for dementia [4]. Among them, lifestyle related diseases such as hypertension, diabetes, and obesity contribute to dementia, especially in middle age. They induce vascular dysfunction, which causes loss of energy consumption by the neuron and results in neuronal dysfunction. Thus, the management of lifestyle-related diseases, such as hypertension, may prevent the onset or progression of dementia through vascular protection.

## Main text

### Influence of mid-life hypertension on dementia

Mid-life hypertension is reported to be a factor inducing dementia. Mid-life is typically defined as 45–64 years old. In this manuscript, mid-life hypertension means hypertension in individuals approximately 50 years old, and late-life hypertension means hypertension in those approximately more than 70 years old. On the other hand, early-life hypertension is meaningless because individuals with hypertension before mid-life could have a risk for the development of dementia. The Atherosclerosis Risk in Communities (ARIC) cohort showed that high blood pressure in mid-life (around 48–67 years old) induces poorer cognitive function or dementia 20 years later [7]. Moreover, the Honolulu Heart Program or Honolulu Asia Aging Study demonstrated that subjects less than 50 year old, even those with prehypertension, had an increased risk for dementia only in the untreated group [8]. Interestingly, subjects receiving antihypertensive medication showed no increased risk for dementia, even in those with systolic blood pressure of more than 140 mmHg, indicating that early intervention in hypertension is one approach to prevent late-life dementia. So, what is the target level of blood pressure in mid-life to prevent dementia? Abell et al. reported that systolic blood pressure elevation at age 50 years is associated with increased risk of dementia [9]. Moreover, a systolic blood pressure level of 130 mmHg or lower has been shown to significantly prevent dementia at age 50. However, blood pressure elevation at 60 or 70 years old is not a significant risk, even in those with severe high blood pressure [9].

### Estimated mechanisms of mid-life hypertension-induced dementia

Mid-life hypertension has been shown to be a risk for vascular dysfunction. Brain aging is considered to be induced not via neural aging, but via dysfunction of a coordinated and interactional system of neurons, astrocytes, and microvessels in the brain called the “neurovascular unit”. A chronic hypertensive state induces cerebrovascular degeneration such as vascular remodeling, vascular hypertrophy, atherosclerosis, endothelial dysfunction and increased blood brain barrier permeability, resulting in disorder of the neurovascular unit [10]. These results indicate that vascular degenerative disease causes neurodegenerative disease such as dementia including Alzheimer’s disease. Therefore, recently Qiu et al. introduced the concept of dementia in the elderly as a condition of total life course events via vascular injury in mid-life, and cardio- or cerebrovascular disease in later life [11].

### Influence of late-life hypertension on dementia

On the other hand, intervention for high blood pressure in the very elderly did not significantly reduce the incidence of dementia in the Hypertension In the Very Elderly Trial-COGnitive function assessment (HYVET-COG) trial [12]. Moreover, the HYVET cohort study demonstrated that orthostatic hypotension indicates an increased risk of dementia and cognitive decline [13]. Thus, intensive BP treatment to prevent dementia is not recommended in “very elderly people” because blood pressure lowering fails to maintain cerebral blood flow because of dysfunction of cerebral autoregulation. Therefore, the younger the age at which blood pressure is managed at an appropriate level the better in order to prevent cognitive decline.

### Renin-angiotensin-aldosterone system and dementia

In the cardiovascular burden over the course of a lifetime, the renin-angiotensin system (RAS) plays an important role as a risk or inducing factor for cardio- and cerebrovascular disease. We have been investigating the effect of RAS on cognitive function using animal models. The effect of angiotensin (Ang) II, a main component of RAS, on cognition is controversial. Gard et al. previously reviewed the dual action of Ang II in memory and learning [14]. For example, Georgiev et al. demonstrated that Ang II facilitates learning and memory [15]. On the other hand, Kulakowska et al. and Barnes et al. showed that blockade of RAS with an Ang II type 1 receptor blocker (ARB) or Ang converting enzyme inhibitor (ACEi) facilitated memory and learning [16–18]. Thus, we investigated the effect of Ang II on learning ability using Tsukuba-hypertensive mice which are double transgenic for human renin and human angiotensinogen [19]. Wild-type (WT) and Tsukuba-hypertensive mice underwent shuttle avoidance test every week from 10 to 20 weeks old. WT mice showed an increase in avoidance rate (which means better cognition) during aging. Ang II-overexpressing mice exhibited improvement of learning ability at a younger age; however, during later age learning ability did not improve but was impaired, indicating that Ang II may facilitate learning temporarily, but continuous stimulation with Ang II may exhaust the neural system or induce damage in the brain, resulting in cognitive decline [20]. We previously reported the effect of diabetes mellitus on cognition using obese diabetic mice, KKAy [21]. Interestingly, RAS blockade by ARB inhibits diabetes-induced cognitive impairment in these mice via prevention of BBB disruption with attenuation of swollen astrocyte end-feet. Ito et al. demonstrated that swollen astrocyte end-feet compress micro-vessels and induce dysfunction of the microcirculation [22]. Ang II-overexpressing mice also showed BBB disruption even in younger mice (unpublished data). Recently, Franco et al. clearly showed the potential mechanisms of hypertension-induced cognitive deficit involving angiotensin II, which increases the superoxide-producing enzyme, NADPH oxidase 2 (NOX2), in perivascular macrophages and induces reactive oxygen species [23]. These basic experiments suggest a protective effect of RAS blockade on cognitive decline.

### Influence of BP control on dementia

Li et al. demonstrated that subjects treated with an ARB exhibit a lower incidence of dementia and an increase in survival probability compared with subjects with other cardiovascular comparators, using the US Veteran Affairs database of 819,491 male subjects [24]. However, to date, BP lowering has not been shown conclusively to preserve cognitive function in a clinical trial [25]. Moreover, intervention for high blood pressure in the very elderly does not significantly reduce the incidence of dementia as described above [12], and orthostatic hypotension is an increased risk for dementia and cognitive decline [13], indicating several problems in the sample age (average age around 70 years) and observation period (less than 5 years). A short observation time and small number of subjects over middle age might affect the results of clinical trials. Further investigation is necessary to confirm the preventive effect of blood pressure lowering on cognition. The American Heart Association has published a scientific statement on hypertension on cognitive function [26]. This states “there is strong evidence of a deleterious influence of mid-life hypertension on late-life cognitive function, but the cognitive impact of late-life hypertension is less clear.” It also states that “evidence from clinical trials that antihypertensive treatment improves cognition is not conclusive.” Recently, the Systolic Blood Pressure Intervention Trial (SPRINT) Memory and Cognition in Decreased Hypertension (SPRINT MIND) demonstrated that intensive blood pressure lowering prevents mild cognitive impairment and possibly dementia and slows development of white matter lesions in the brain [27]. Average blood pressure was 121.4 mmHg in the intensive-treatment group and 136.2 mmHg in the standard treatment group. Thus, in the future more clinical trials are needed to provide evidence that “the lower the blood pressure, the lower the incidence of dementia”.

### Recommendations for management of blood pressure to prevent dementia

**Fig. 1:**
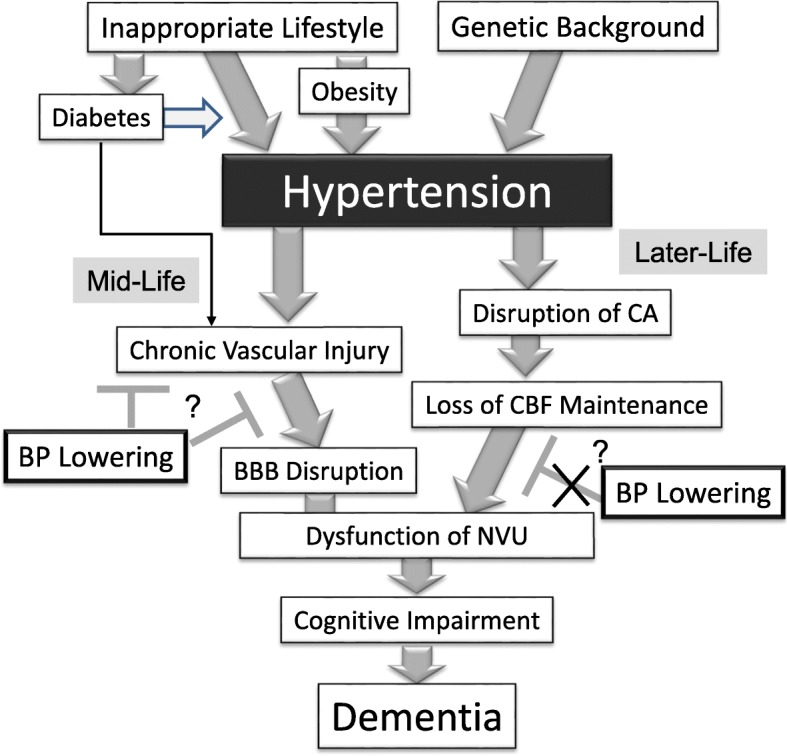
Relation between hypertension and dementia. BBB; blood brain barrier, BP; blood pressure, CA; cerebral autoregulation, CBF; cerebral blood flow, NVU; 18 neurovascular unit

A summary of this review is presented in Fig. 1. Current recommendations for the management of blood pressure to prevent dementia are as follows:

1. Attention to untreated subjects with BP of more than 120/80 mmHg for vascular health.
2. Subjects with systolic BP of more than 130 mmHg without medication (mid-life hypertension) have increased future risk of dementia.
3. More attention is needed in subjects with other lifestyle risks.
4. There is no evidence that antihypertensive treatment can reduce the incidence of dementia, to date.
5. BP in “aged people” should be carefully managed, and BP lowering has no conclusive preventive effect on dementia in the very elderly.
6. However, the best way to prevent dementia in hypertensive patients is intensive treatment from middle age.

## Conclusions

Management of hypertension from middle age may reduce the onset of dementia in the elderly. However, large clinical trials are necessary to confirm whether antihypertensive drugs prevent dementia.

## Data Availability

Not applicable.

## Abbreviations

AD: Alzheimer’s disease
Ang II: Angiotensin II
ARB: Angiotensin II type 1 receptor blocker
BBB: Blood brain barrier
RAS: Renin-angiotensin system
VD: Vascular dementia
